# Strong activation effect on a Ru-Co-C thin film catalyst for the hydrolysis of sodium borohydride

**DOI:** 10.1038/s41598-018-28032-6

**Published:** 2018-06-27

**Authors:** G. M. Arzac, M. Paladini, V. Godinho, A. M. Beltrán, M. C. Jiménez de Haro, A. Fernández

**Affiliations:** 1 Instituto de Ciencia de Materiales de Sevilla (CSIC-Univ. Sevilla), Avda. Américo Vespucio 49, 41092 Sevilla, Spain; 2 Departamento de Ingeniería y Ciencia de los Materiales y del Transporte, Universidad de Sevilla, Escuela Politécnica Superior, Virgen de África 7, 41011 Sevilla, Spain

## Abstract

In this work, we prepared a series of Ni foam supported Ru-Co, Ru-Co-B and Ru-Co-C catalysts in the form of columnar thin films by magnetron sputtering for the hydrolysis of sodium borohydride. We studied the activity and durability upon cycling. We found a strong activation effect for the Ru-Co-C sample which was the highest ever reported. This catalyst reached in the second cycle an activity 5 times higher than the initial (maximum activity 9310 ml.min^−1^.g_CoRu_^−1^ at 25 °C). Catalytic studies and characterization of the fresh and used samples permitted to attribute the strong activation effect to the following factors: (i) small column width and amorphous character (ii) the presence of Ru and (iii) dry state before each cycle. The presence of boron in the initial composition is detrimental to the durability. Our studies point out to the idea that after the first cycle the activity is controlled by surface Ru, which is the most active of the two metals. Apart from the activation effect, we found that catalysts deactivated in further cycles. We ascribed this effect to the loss of cobalt in the form of hydroxides, showing that deactivation was controlled by the chemistry of Co, the major surface metal component of the alloy. Alloying with Ru is beneficial for the activity but not for the durability, and this should be improved.

## Introduction

For a sustainable future, it is necessary an energy transition which decreases the amount of greenhouse gases released to the atmosphere to mitigate the climate change. In this context, hydrogen (H_2_) is a very interesting energy vector which generates energy with water as only by product^1^. For the implementation of the hydrogen as alternative fuel, it is necessary to overcome numerous barriers such as sustainable and low cost production, transportation and storage^1–3^. Sodium borohydride (NaBH_4_, SB) is a safe, stable and low-weight hydrogen storage material with a high hydrogen storage capacity 10.9 wt.%^4–6^. Its hydrolysis reaction (NaBH_4_ + 2 H_2_O → 4H_2_ + NaBO_2_), is exothermic, being able to generate hydrogen even at low temperatures^4–6^. The hydrogen produced is free from carbon monoxide, being able to supply a Polymer Exchange Membrane Fuel Cell with no further purification steps.

In order to produce the hydrogen at appreciable rates, it is necessary the addition of appropriate catalysts. Heterogeneous metal supported catalysts are the most reported for this reaction^7^. Precious metal catalysts such as Pt, Rh, Pd and Ru usually exhibit better performance than the non-noble ones, but they are less abundant and thus more expensive^4,8–11^. Among the non-noble metal catalysts, cobalt-based are the most investigated because of their cost-efficiency ratio and abundancy^12^, although they show a high tendency to deactivate upon use^13,14^. A very efficient strategy to increase the activity of Co based catalysts is alloying with other metals. The performance of the alloy is usually better compared to the monometallic catalysts^15^. In particular, the use of Co-Ru bimetallic alloys is advantageous, because the activity of Co can be increased with the addition of small amounts of Ru, with less impact in the cost than by adding Pt, Pd and Rh, which are more expensive^16–20^.

In our previous reports we employed Magnetron Sputtering (MS) as a method to prepare thin film catalysts for the hydrolysis of sodium borohydride. The MS is ideal for the study of structure-performance relationships because the microstructure and composition can be tuned on demand by varying the deposition conditions. We prepared a series of Ni foam supported pure Co^21^, Co-B and Co-C^22^ thin film catalysts and studied their activity and durability. Under optimized conditions, the maximum activity achieved was 2685 ml.min^−1^g_catalyst_^−1^ for a Co-B sample. With the aim to go a step forward and improve the activity we decided to work with bimetallic Ru-Co samples with 13 at. % Ru (respect of total metal). This composition was chosen as a compromise between activity and cost, according to our previous investigation on powdery catalysts^18^.

In this paper, we have prepared a series of Ni foam supported Ru-Co, Ru-Co-B and Ru-Co-C thin films by MS to be used as catalysts for the hydrolysis of SB. We have investigated the role of Ru, Co and B in the activity and durability upon cycling. Besides, we have studied the activation effect occurring in the three samples, which activity increased within the first cycles. Finally we discuss the requirements for this type of catalysts to achieve the upon use strongest activation every reported, which were fulfilled herein by the Ru-Co-C sample.

## Results and Discussion

### Characterization

**Figure 1:**
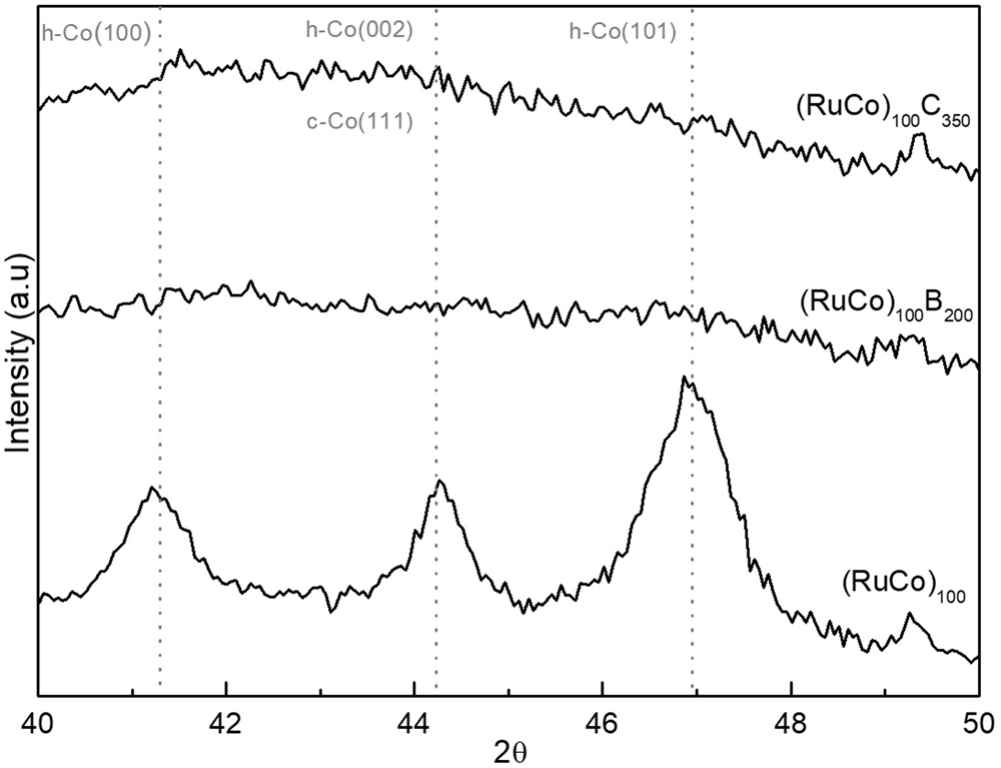
XRD measurements on the prepared samples.

Figure 1 shows the XRD (X-ray diffraction) measurements for the catalysts supported on PTFE (polytetrafluoroethylene) membranes. The (RuCo)_100_ sample shows small and broad peaks which can be assigned to a nanocrystalline hcp Co phase (ICDD 00-005-0727), although the cubic phase (ICDD 00-015-0806) cannot be disregarded. The incorporation of B and C produces amorphization of catalysts as demonstrated by the absence of peaks for the carbon and boron containing samples^22^.

**Figure 2:**
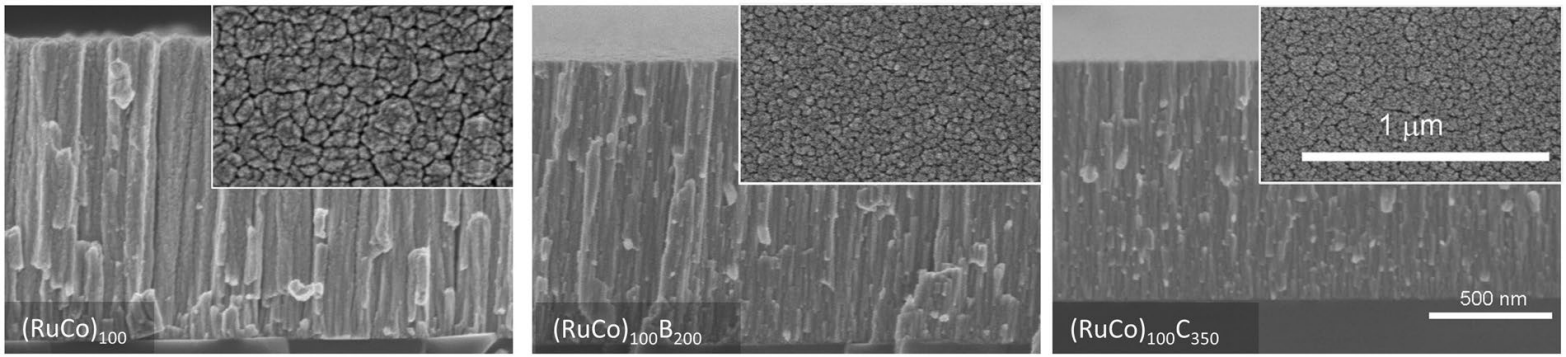
Cross sectional and top view (inset) SEM images on prepared samples. Scale bars are the same for cross and top views respective images.

**Figure 3:**
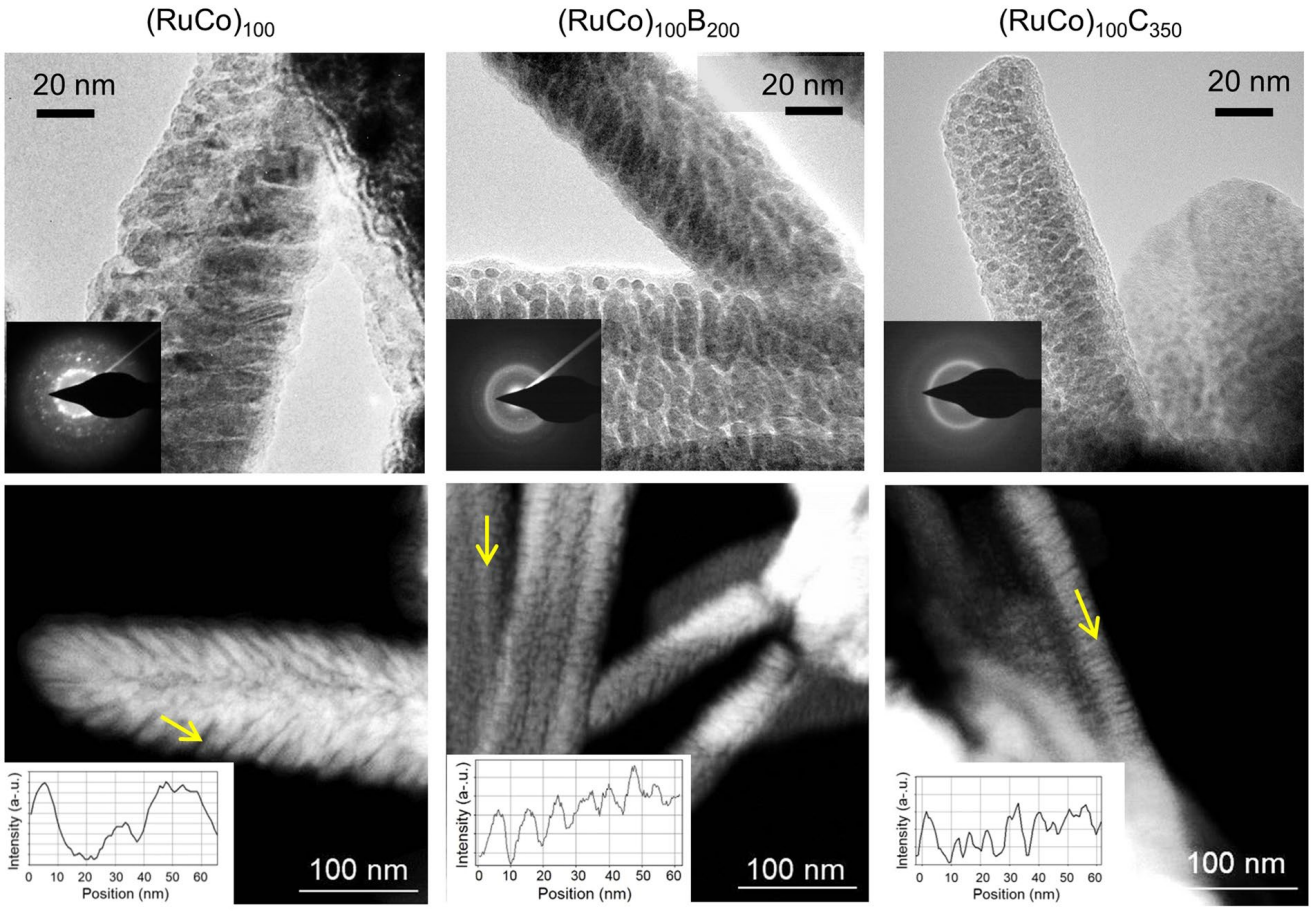
TEM (top) and STEM/HAADF (bottom) images on the as-prepared samples. Insets: SAED (top) and HAADF intensity profiles as a function of the position (bottom).

Figure 2 shows the representative SEM (Scanning electron microscopy) top and cross sectional views of the coatings. The catalysts present a columnar growth, typical of thin films deposited by magnetron sputtering under low adatom mobility conditions, where surface shadowing governs the film growth^23^. This characteristic microstructure consists on the formation of mesocolumns constituted in turn by nanocolumns. The top views clearly show that the (RuCo)_100_ sample has thicker mesocolumns than the others (see width range data in Table 1). The cross section views permit to determine the thin film thickness (1–1.2 μm) and the deposition rate (Table 1). Representative TEM (Transmission Electron Microscopy) and STEM/HAADF (scanning TEM/ High Angle Annular Dark Field) images of the PTFE supported catalysts are in Fig. 3. These images reveal intra and inter-columnar porosity. The SAED (Selected Area Electron Diffraction) patterns in Fig. 3 show higher crystallinity for the (RuCo)_100_ sample, while the addition of B or C produces amorphization in accordance to XRD data. The sample (RuCo)_100_C_350_ shows the highest amorphization degree. The nanocolumn width ranges in Table 1, calculated from the study of a series of intensity line profiles (with a width of 10–15 nm) on STEM/HAADF images (representative HAADF intensity line profiles in Fig. 3), confirm that the (RuCo)_100_ sample has the highest nanocolumn width (9–15 nm), followed by the (RuCo)_100_B_200_ (5–8.5 nm) and the (RuCo)_100_C_350_ (3–6.5 nm). Smaller nanocolumn width correlates with higher active surface area and thus better catalyst dispersion as demonstrated before^21^.

**Table 1:** Deposition conditions and microstructural parameters for the as-prepared catalysts.

Catalyst	Deposition time (min)	Ar pressure (10^−2^ mbar)	CoRu power (W)	B or C power (W)	Thickness (nm) (by SEM)	Deposition rate (nm.min^−1^)	Mesocolumn width (nm) (by SEM)^(a)^	Nanocolumn width (nm) (by TEM)^(a)^
(RuCo)_100_	120	2.8	100	—	1190	9.9	50–140	9.0–15.0
(RuCo)_100_B_200_	120			200	1160	9.7	40–80	5.0–8.5
(RuCo)_100_C_350_	90			350	1000	11.1	50–75	3.0–6.5

^(a)^Representative column width ranges.

**Figure 4:**
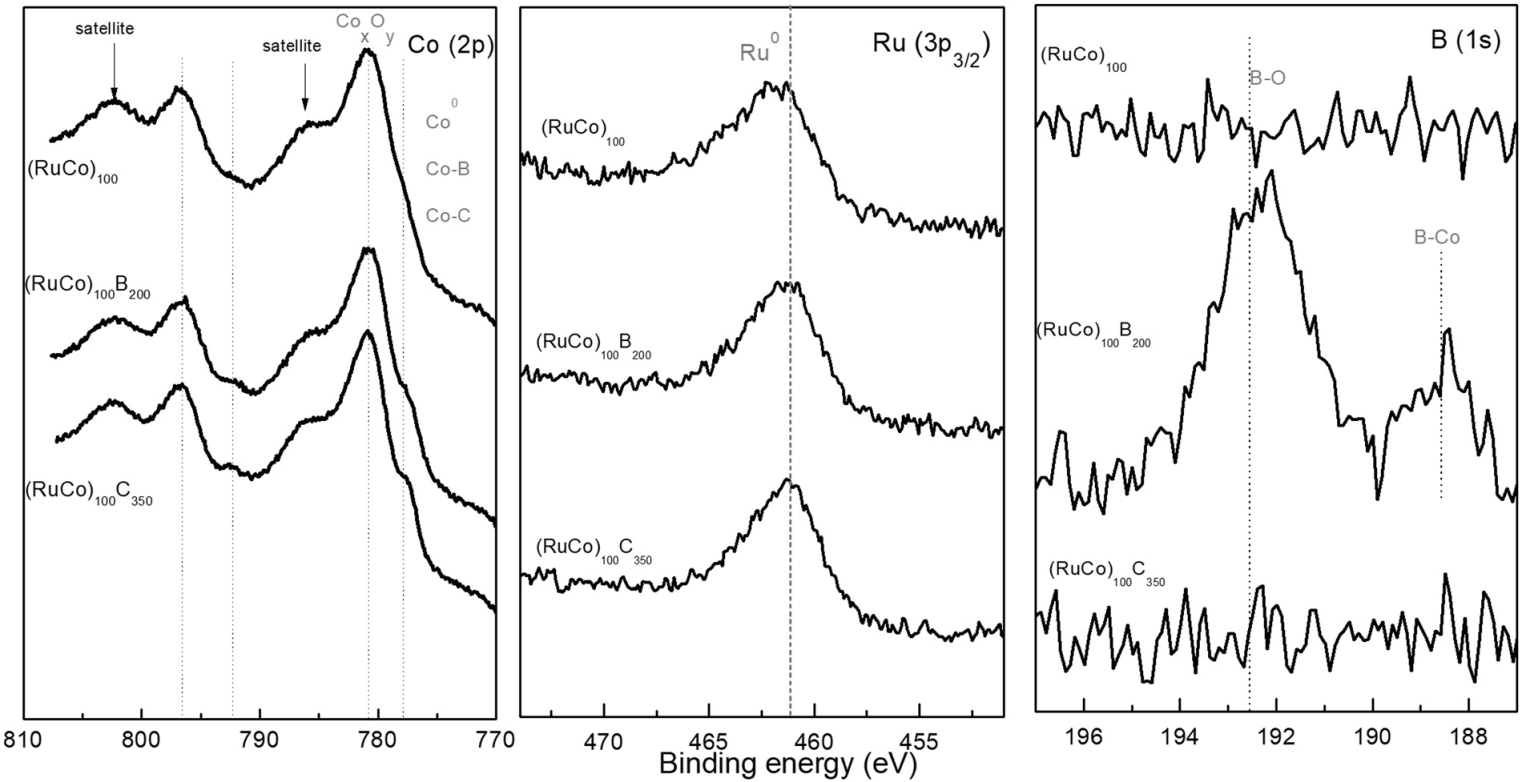
Normalized XPS spectra on the as-prepared samples.

Surface electronic states were studied by XPS (X-Ray Photoelectron Spectroscopy) and Fig. 4 shows the normalized spectra. The study of the Co (2p) level shows the presence of oxidized cobalt at 780.9 eV (Co2p_3/2_) and 796.8 eV (Co2p_1/2_) for all the samples^24^. The shape of peaks and satellites is characteristic of the Co^II^ state. Boron and carbon containing samples also show the signals at 777.8 (Co 2p_3/2_) and 792.9 eV (Co 2p_1/2_) characteristic of metallic cobalt, cobalt carbides or cobalt borides^22,24^. The analysis of the B 1s level shows the presence of the two peaks at binding energies of 188.5 and 192.4 eV attributed to elemental boron or boron in Co_x_B compounds and oxidized boron respectively^24^. The Ru 3d level is difficult to quantify because of the superimposition with the C 1s. Therefore we have also measured the Ru 3p_3/2_ level, which indicates the presence of metallic Ru in the three samples, characterized by the peak position at 461.1 eV^25^.

Proton-elastic back-scattering spectrometry (p-EBS) was employed to calculate bulk composition and results are shown in Table 2. For the three samples, the molar fraction of Ru (x_Ru_ = n_Ru/(_n_Ru_ + n_Co)_) respect to total metal is 0.13–0.14 in agreement with the target composition (13% at. Ru), indicating that the sputtering process was equally efficient for both elements. The (RuCo)_100_ sample has the highest amounts of oxygen (14.6 at.%) followed by the (RuCo)_100_B_200_ and the (RuCo)_100_C_350_. The amount of boron and carbon incorporated is similar (30 and 35 at.% for the (RuCo)_100_C_350_ and (RuCo)_100_B_200_ respectively), showing that the MS method is efficient in controlling the sample composition by taking into account the target power and the sputtering yield at the depositing conditions.

**Table 2:** Bulk and surface composition of the prepared catalysts.

Catalyst	Bulk composition (p-EBS)								Surface composition (XPS)				
	at. % Co	at. % Ru	at. % C	at. % B	at. % O	at. % total metal	wt.% total metal	x_Ru_	at. % Co 2p	at. % Ru 3p_3/2_	at. % B 1 s	at. % O1s	x_Ru surface_
(RuCo)_100_	73.2	12.2	—	—	14.6	85.4	96.0	0.14	28.4	3.6	—	68	0.11
(RuCo)_100_B_200_	50.3	7.7	—	35.0	7.0	58.0	88.0	0.13	23.3	2.4	10.0	64.3	0.09
(RuCo)_100_C_350_	57.5	9.5	30.2	—	2.9	67.0	91.0	0.14	26.9	3.3	—	69.8	0.11

XPS spectra in Fig. 4 were quantified and results are shown in Table 2. The three samples show high amounts of total surface oxygen 69.8–64.3% as expected for these catalysts exposed to ambient air conditions. Considering the presence of reduced cobalt in (RuCo)_100_B_200_ and (RuCo)_100_C_350_ and oxidized boron in the (RuCo)_100_B_200_ we can affirm that oxygen is strongly bound to boron and carbon, protecting the metals atoms from oxidation^19^. Calculation of the molar fraction of Ru respect to total metal (around 0.10–0.11) demonstrated that the relative metal composition is similar in the surface than in the bulk for the three samples, within the experimental error.

### Catalytic activity

#### Initial activities

The initial activity of the catalysts supported on Ni foam was measured at 25 °C. The hydrogen evolution curves follow a straight line indicating zero-order kinetics in SB concentration (Figure S1, as supporting information). Despite samples contain surface oxygen as demonstrated by XPS, no induction period was detected in the experiments. It means that the reduction of the catalyst surface under contact with SB occurred very rapidly^26^. Activities (in ml.min^−1^ . g_catalyst_^−1^) were obtained from the plot of the hydrogen generation rates as a function of the mass of catalyst (Figure S1, as supporting information). By taking into account the wt.% of total metal obtained by p-EBS (Table 2), we were able to calculate the activity in ml.min^−1^.g_Co+Ru_^−1^, and the results are shown in Table 3. The activity trend is (RuCo)_100_B_200 _> (RuCo)_100_C_350_ > (RuCo)_100_. The lower activity of the (RuCo)_100_ can be explained by its higher column width and crystalline degree^22^. The higher activity the (RuCo)_100_B_200_ is attributed to the presence of boron in the composition which produces electronic effects^22^, despite the (RuCo)_100_C_350_ sample is the most disperse of the three (smaller column width).

**Table 3:** Catalytic results on the prepared samples.

Catalyst	Initial activity (ml.min^−1^.g_Co+Ru_^−1^)	Ea (kJ.mol^−1^)	Thorough washing	Quick washing
			Maximum activity (ml.min^−1^.g_Co+Ru_^−1^)	Maximum activity (ml.min^−1^.g_Co+Ru_^−1^)
(CoRu)_100_	1115	53	1547	4570
(CoRu)_100_B_200_	1873	66	3000	3062
(CoRu)_100_C_350_	1560	64	7228	9310

The activity of the samples was measured as a function of temperature and the Arrhenius plots are shown in Figure S1. The apparent activation energies (AAEs) are shown in Table 3. The (RuCo)_100_ sample has the lowest and both (RuCo)_100_B_200_ and (RuCo)_100_C_350_ have similar AAE, probably in accordance with the similar microstructure.

#### Maximum activities

**Figure 5:**
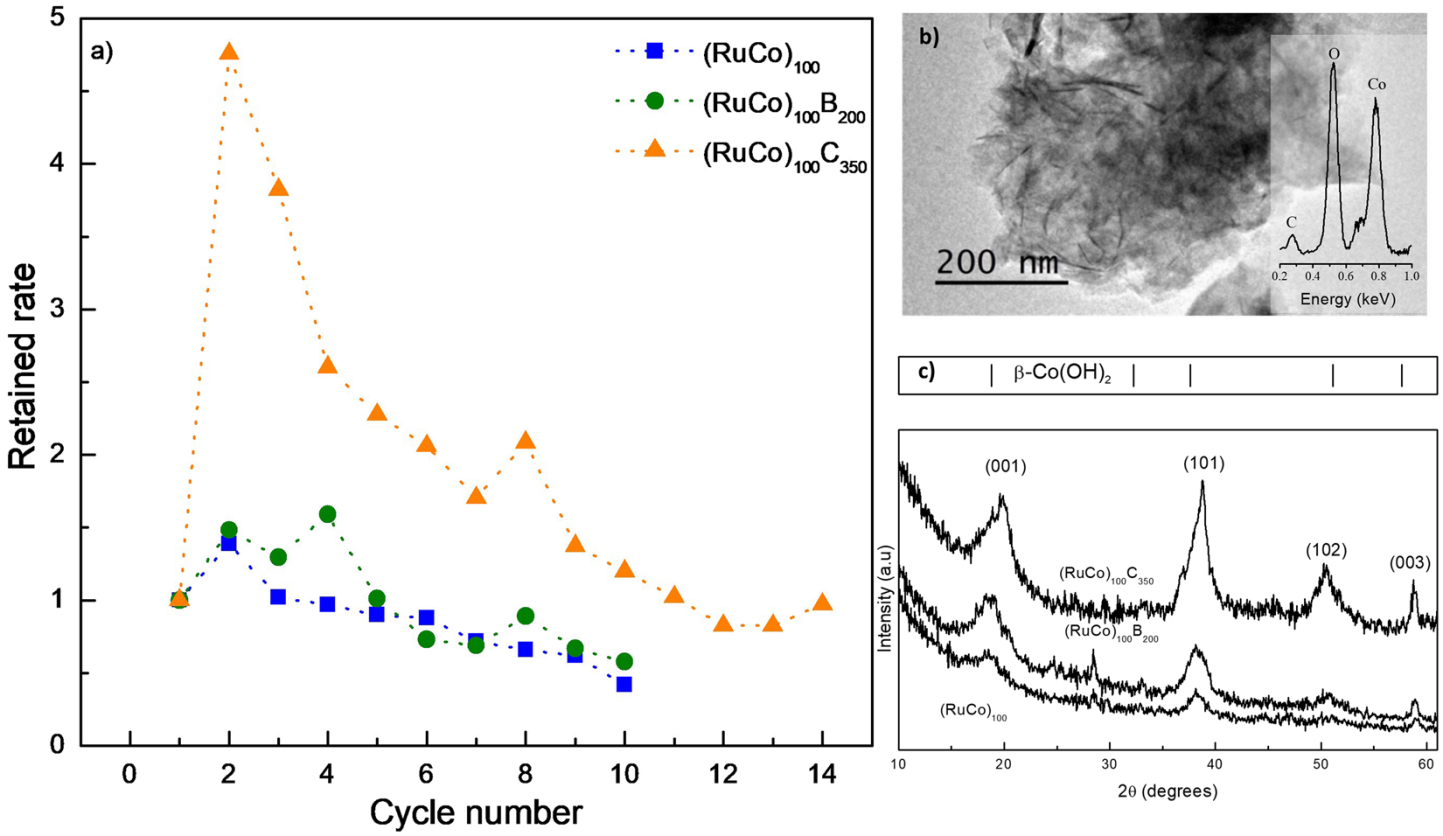
Activity and durability of the prepared samples upon cycling with thorough washing and drying between cycles. (**a**) Retained rate (HGR/HGR _initial_) as a function of the cycle number. (**b**) Representative TEM image of the precipitates obtained in the supernatant and washing solutions upon cycling (inset: EDX spectrum). (**c**) XRD measurements of the precipitates obtained in the supernatant and washing solutions upon cycling.

The activity of the prepared samples was measured upon cycling at 25 °C. Each cycle was performed daily, and after each one, the catalyst was extracted, thoroughly washed and dried at RT overnight. Figure 5 shows the plot of the retained rates as a function of the cycle number. For the three catalysts, the activity is first increased and then decreased. This increase in the activity upon use (activation effect) was reported for the first time in our previous studies on Co-B and Co-C catalysts, but not higher than 2 times respect to the initial^22^. In the present work, the magnitude of the activation effect for the (RuCo)_100_C_350_ is the highest ever reported for this reaction. The activity of the catalyst in the second cycle is almost 5 times higher than the initial one reaching a maximum of 7228 ml.min^−1^.g_CoRu_^−1^.

**Figure 6:**
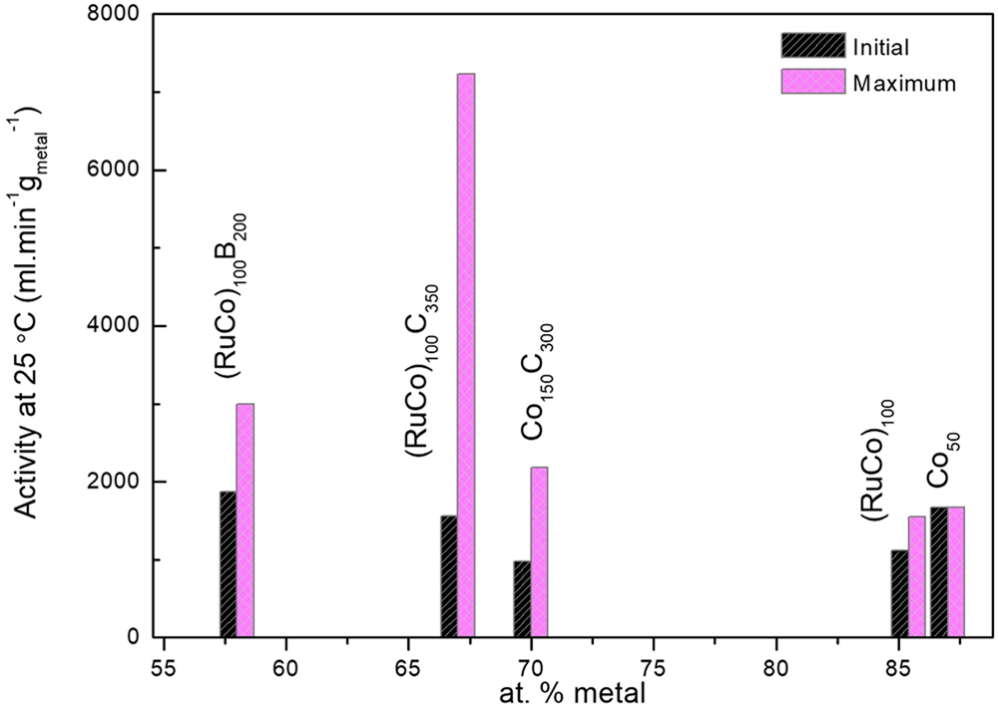
Maximum and initial activities of the prepared samples as a function of the at.% of total metal (p-EBS), in comparison with selected catalysts (Co_50_ and Co_150_C_300_) prepared in our previous works.

The maximum activities (Table 3) were plot together with the initial ones as a function of the at.% of total metal obtained by p-EBS (Fig. 6). The maximum activity trend is (RuCo)_100_C_350_>> (RuCo)_100_B_200_ > (RuCo)_100_ This trend correlates with the higher active surface area or dispersion associated to the smaller nanocolumn width and higher amorphous character according to our previous observations^22^.

**Figure 7:**
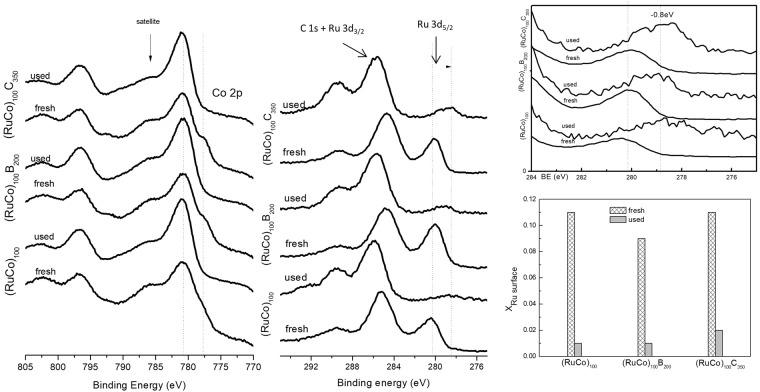
Normalized XPS spectra of the used catalysts in comparison with the fresh ones. At the bottom-right: surface composition of the used catalyst in comparison with the fresh ones (x_Ru surface = nRu surface_/_(nRu surface + nCo surface)_).

To study the role of Ru in the activation effect, we compared the Ru-Co samples prepared in this work with our previously reported Co catalysts of similar crystallinity, column width and metal composition (metal at.%, p-EBS)^22^. In this context, Co_150_C_300_ may be directly comparable with (RuCo)_100_C_350_ and Co_50_ may be directly comparable with (RuCo)_100_. The activities are also plot in Fig. 6. At similar metal composition, the activation effect observed for the Ru containing samples is higher than those with no Ru. For a better understanding of the phenomenon, we measured the XPS spectra of the samples after use and compared them with the fresh ones. Figure 7 shows the results. The study of the Co 2p level shows that after use, the surface cobalt becomes oxidized, with no contribution of Co°, Co-B or Co-C signals. This is in agreement with previous observations indicating the formation of cobalt hydroxides, oxides and borates on catalyst surface after use^27^. We studied the Ru 3d_5/2_ signal which does not overlap with the C1s level. We observed that after use, the intensity of the Ru 3d_5/2_ is significantly decreased. Normalized spectra show however a negative shift in the position of Ru^0^, indicating an electron transfer to Ru for the used samples (see Fig. 7 top-right). No boron was detected on the surface of the used samples. Quantification was performed using the Co 2p and Ru 3p_3/2_ levels and results are shown in the same figure (Fig. 7 bottom-right). The comparison of the fresh with the used catalysts shows that the amount of surface Ru is significantly decreased upon use. Co surface segregation occurs, probably induced by the presence of hydroxide and borate anions in the solution (adsorbate-induced surface segregation)^18,28,29^. The catalyst surface is thus mostly composed of cobalt. The amount of surface Ru may however play an important role producing synergistic effects^17,18^. The shifts to lower binding energy of the Ru peaks upon catalysts use indicate an increased dispersion of Ru, thus explaining the higher relative activation for the Ru-containing samples in comparison to pure Co samples. Both Co and Ru contribute to the measured activity, but the latter, being most active than the former, contributes in a higher degree.

#### Deactivation and activation effects

In the previous section, we rationalized the strong activation effect occurring in the series of Ru-Co samples in terms of column width, crystal size and catalyst composition. We found that to get maximum activation, samples should: i) have small column width and amorphous character and ii) contain Ru. These requirements were fulfilled by the (RuCo)_100_C_350_ catalyst, showing a 5-fold increase in the activity in respect to the initial value upon cycling.

In addition to the activation effect, we found that catalysts deactivated upon successive cycles, as shown in Fig. 5. In our previous work we found that a Co catalyst deactivated through the elimination of cobalt hydroxides during the extraction and washing procedures between cycles^27^. For this reason, in this work, we also studied the sum of the supernatant and washing solutions after each cycle. This study was facilitated by the supported nature of the catalysts. After filtering, we found the presence of brown precipitates which we characterized. XRD measurements in Fig. 5c show the presence of β-Co(OH)_2_ (ICDD 00-030-0443) as major crystalline component of the precipitates obtained from the three catalysts. No peaks corresponding to Ru-based species were found. We also studied the precipitates by TEM and results are also shown in Fig. 5b. A representative micrograph shows the presence of acicular particles as major components of the precipitates, consistent with the presence of β-Co(OH)_2_, as confirmed by the EDX (Energy Dispersive X-Ray Spectroscopy) measurements^27^. No Ru was found in the acicular particles, but a certain amount of Ru was measured by EDX in zones composed by fresh thin film mechanically detached from the support, probably by the action of the evolving H_2_ during the reaction (Figure S2 as supporting information)^27^. All these measurements indicate that the main deactivation mechanism is the elimination of Co in the form of oxidized species. This result is in accordance with surface composition significantly enriched with Co. Similar relationship between the nature of the deactivation mechanism and the identity of major surface component of the alloy was reported for Cu-Co catalysts, but in that case, deactivation was attributed to the adsorption of borates on Co, which was surface segregated respect to Cu^29^.

**Figure 8:**
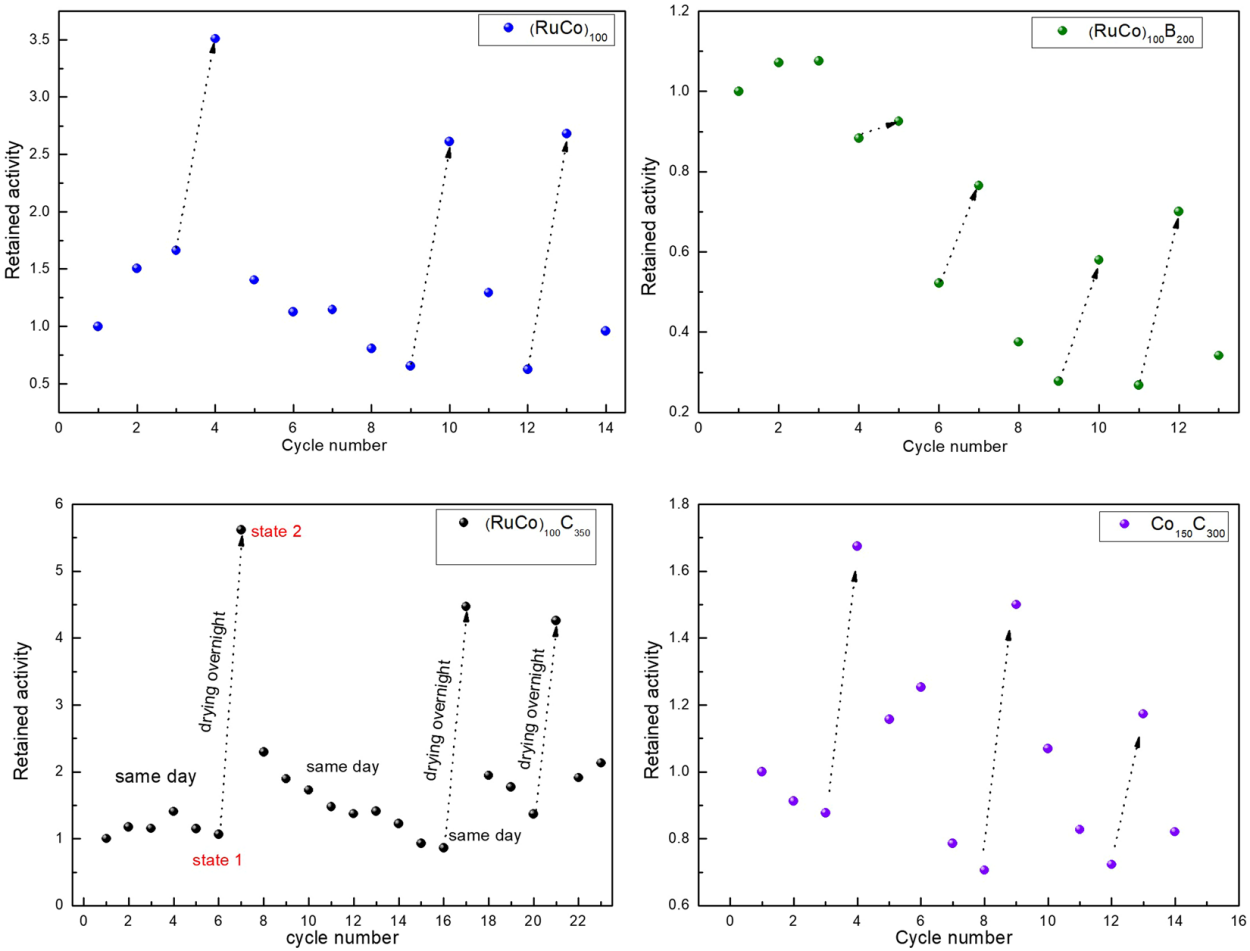
Durability of the prepared samples upon cycling with quick washing and drying effect at some particular cycles. Retained rate (HGR/HGR_initial_) as a function of the cycle number.

In order to gain better understanding of the activation and deactivation effects, we repeated the cycling experiments but under different conditions. In the previous sub-section, the cycling experiments were performed one per day, with extraction, thorough washing and drying of the catalyst between cycles. This procedure favors the loss of cobalt in the form of hydroxides and oxides^27^. To avoid the loss of cobalt, we performed the cycling experiments in the following manner (quick washing): after each cycle, the supernatant solution was extracted and 1 ml of pure water was added. This water was extracted and stored with the supernatant solution. Some cycling experiments were performed in the same day, and some after catalyst drying at RT overnight. Under no circumstance, the catalyst was extracted from the reactor, to avoid excessive manipulation. The cycling experiments were performed on the Ru-Co-(B,C) samples of this work and for a Co_150_C_300_ prepared before^22^, to understand the role of Ru. Figure 8 shows the results. The arrows indicate overnight drying, and the points between arrows were performed within the same day, with no catalyst drying.

The plot of the retained activity as a function of the cycle number shows that all catalysts first activate and then deactivate upon use. For cycles performed within a single day, the behavior is a first small or no activation and then continuous deactivation. However, after catalyst drying, a strong activation occurs. The magnitude of the activation is within the order of magnitude of the one obtained in cycles with thorough washing and drying between cycles (Fig. 5). These results permit to ascribe the strong activation to a dry catalyst and not to a wet one. The magnitude of the activation is still the highest for the (RuCo)_100_C_350_ sample. The (RuCo)_100_B_200_ catalyst activates upon drying, but the overall behavior is a continuous decrease in the activity respect to the initial with use, indicating higher tendency to deactivation. This shows that the presence of boron in the initial composition is not detrimental for activation but favors deactivation. The maximum activities obtained with the quick washing-drying procedure are shown in Table 3. The value for the (RuCo)_100_C_350_ sample is 9310 ml.min^−1^.g_CoRu_^−1^. Comparing with another catalysts of similar composition reported in literature (Table 4, in ml.min^−1^.g_catalyst_^−1^), the performance of the thin film is higher than the one obtained with powdery Ru-Co-B^18^ and Ru-Co/C^20^ materials (the latter with less amount of Ru than herein). The use of Ru and Co metal salts as precursors to *in-situ* generate a Ru-Co catalyst (of exactly the same composition than herein) permits to achieve significantly higher activities, but with a decrease in the performance with time because of catalyst aggregation^16^. Respect to the maximum activities obtained with Co-based thin films in the past, the improvement achieved in this work is significant^21,22^. However, additional work should be conducted in order to improve the durability of the samples for the implementation of these catalysts in a practical application.

**Table 4:** Comparison with other catalysts in literature.

Catalyst	Initial activity (ml.min^−1^.g_catalyst_^−1^)	Maximum activity (ml.min^−1^.g_catalyst_^−1^)	T (°C)	Reference	Form
(RuCo)_100_C_350_	1420	8472	25	This work	thin film
RuCo/C	1550	—	25	ref.^20^	powder
Ru-Co-B	3158	—	25	ref.^18^	powder
Ru-Co	17200	—	25	ref.^16^	Powder (*in-situ* generated)

**Figure 9:**
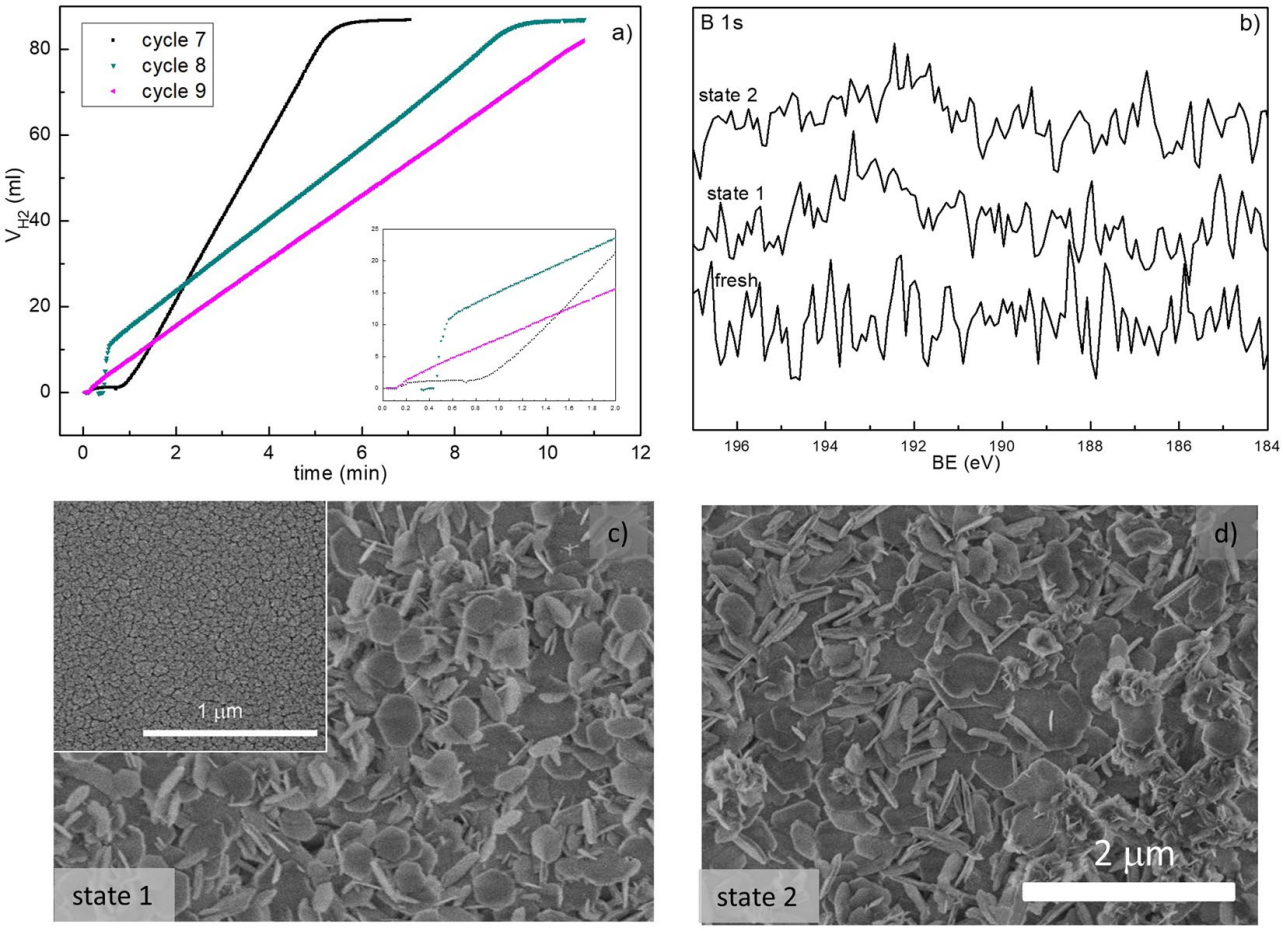
Study of the strong activation effect for the (RuCo)_100_C_350_ sample. (**a**) Hydrogen evolution curves for the 7^th^–9^th^ cycles. (**b**) XPS spectra for the catalysts before and after the 7^th^ cycle (states 1 and 2 respectively). Representative SEM images of the catalysts before (**c**) and after (**d**) the 7^th^ cycle (inset: fresh catalyst).

For a study of the strong activation process occurring for the (RuCo)_100_C_350_ catalyst in dry form, the sample was studied before (state 1) and after the 7^th^ cycle (state 2), which showed the high activation (see Fig. 8). The cycles were performed on the Ni foam supported catalyst in presence of 1 cm^2^ of the same material but PTFE supported for characterization. Hydrogen evolution curves of the 7^th^–9^th^ cycle are depicted in Fig. 9a. The 7^th^ cycle shows a 1 min activation period but after this, the hydrogen generation rate is the highest of the three. The 8^th^ cycle shows a shorter activation period (around 0.5 min) and the 9^th^ cycle shows no detectable activation, both cycles with decreasing hydrogen generation rates. These differences in the length of the activation period and in the activity are clearly indicative of the formation of an active metallic phase with markedly different properties (particle or grain size, amorphous character, composition, metal reducibility, metal dispersion, etc) in each case. The dry state is thus favoring the *in-situ* formation of a very active metallic phase upon contact with SB.

In order to get some evidence of the microstructure and composition of this very active metallic phase, the PTFE catalyst was cut into two pieces and was characterized after the 6^th^ cycle (state 1, before the strong activation) and after the 7^th^ cycle (state 2, after the activation). The pieces needed however to be extracted, thoroughly washed and dried before characterization by SEM and XPS due to vacuum requirements of both techniques and to avoid the residues of reaction media to cover the surface of the samples. In consequence, the wet state after the 7^th^ cycle (the real state before the 8^th^ cycle) cannot be investigated with these techniques and neither by TEM. In spite of this, the comparison of representatives SEM images of the states 1 and 2 with the fresh one shows that after use, the catalyst surface is covered by precipitates, as occurred for other Co catalysts^27^. The SEM images in Fig. 9c do not reveal any structural difference between the samples before and after the 7^th^ cycle. The study of the Co and Ru XPS spectra (not shown) in comparison to the ones from the fresh sample reveal the oxidation of surface Co and the antisegregation of surface Ru upon use as also shown in Fig. 7. The study of the B 1 s level in Fig. 9b does not show the contribution of surface boron. This result is very important, because deactivation processes are usually attributed to the presence of borates^13,14,30,31^. In our conditions, neither activation nor deactivation can be conclusively explained by the incorporation of boron on catalyst surface. Our previous studies demonstrated that Co-C catalysts incorporated boron in the form of borides and borates upon use, suggesting the *in-situ* formation of Co_x_B as active phase during the reaction^22^. The absence of borides (or borates which come from the decomposition of borides once SB is consumed) in the XPS is indicating that the activity is controlled by the Ru, because this metal does not incorporate much boron in the form of borides upon contact with SB^18,19^. All these results point to the idea that the third requirement to achieve high activation of the (RuCo)_100_C_350_ sample is to be in a dry form. For further comprehension of the phenomenon, samples should be characterized *in-situ* during the 7^th^ cycle and after this cycle in wet form, what is a challenging future work due to experimental limitations of the characterization techniques (SEM and XPS). Previous studies on the activity of Co based catalysts formed *in-situ* by the interaction with SBH of cobalt precursors of different nature could give a plausible explanation of the drying effect^26,32,33^. Co catalysts prepared using metal salts are usually less active than those formed using cobalt oxides as precursors^26,32,33^. The reason is that for metal salts, activation (which is exothermic) is faster and with more rapid heat release than for the oxides, leading to catalyst aggregation and thus decreasing the activity^33^. The activation of oxides occurs slowly because of the low concentration of Co^2+^ in solution^26,32,33^. In our case, the metallic active phase is not only Co-containing but also contains Ru, but the explanation is similar. The activation of the dry precursor takes longer time but the *in-situ* formed metallic particles are more active than those formed from the wet precursor, which contains metal oxides in colloidal state. The latter activate faster but then become aggregated soon^16^. The characterization of these particles is only achievable with *in-situ* techniques, and this is outside of the scope of this work.

## Conclusions

In the present work, we have rationalized the activity of a series of Ru-Co, Ru-Co-B and Ru-Co-C catalysts prepared as thin films by magnetron sputtering. The preparation method permitted to control the microstructure and composition of the catalysts for fundamental structure-performance studies on the hydrolysis of sodium borohydride.

Catalytic studies upon cycling and characterization of the fresh and used states permitted to understand the role or Ru, Co and B in the activity and durability of the samples. A strong activation effect was reported for the first time occurred in the Ru-Co-C sample which activity in the second cycle increased 5 times respect to the initial.

The study of used samples shows a Ru surface antisegregation effect and a shift of the Ru 3d_5/2_ signal to lower binding energies. The antisegregation produces less amount of surface Ru, but more disperse and with higher electron density, which explains the increase in activity. Despite surface is mostly composed of Co, the absence of boron signals coming for the *in-situ* formation of Co_x_B permit to conclude that after the first use, the activity is controlled by Ru. Apart from the activation effect, we found that catalysts deactivated in further cycles. We ascribed this effect to the loss of cobalt in the form of hydroxides, showing that deactivation was controlled by the chemistry of Co, the major surface metal component of the alloy. Alloying with Ru has demonstrated to be beneficial for the activity but detrimental for the stability in cycles.

The activity was measured upon cycling in two conditions: (i) with thorough washing and drying between cycles and ii) with quick washing in wet and dry form. The comparison of the results obtained under the two conditions permitted to conclude that dry catalysts activate in a higher degree than the wet ones. This is because the activation proceeds more slowly preventing further aggregation of catalysts.

To sum up, in this work we found the conditions for a columnar RuCo-based thin film catalyst to achieve high activation which are the following: i) low column width and amorphous character ii) the presence of Ru and iii) dry state before each cycle. The presence of boron in the initial composition favors deactivation and should be avoided. The activation effect was controlled by the antisegregation of Ru, and the deactivation by the unstability of Co against the corrosive reaction medium. Surface segregation effects should be taken into account for the design of the next generation alloy catalysts with improved activity and durability.

## Methods

### Catalyst preparation

The thin film catalysts were prepared by co-deposition and two magnetron sources from AJA for 2′ diameter targets were employed. One was operated in magnetic target configuration under DC power for the RuCo target (13% at. Ru, Angstrom Sciences Inc. 99.95% pure, 1 mm thick). The second one was operated, when appropriate, under RF configuration for pure boron or carbon targets. The base pressure before deposition was 10^−6^ mbar, and working pressure of Ar was 2.8 × 10^−2^ mbar during deposition. Table 1 shows the deposition conditions and the abbreviated names used for the samples, indicating the applied power to the corresponding targets.

For catalytic studies thin films were deposited on commercial Ni foam (Goodfellow 1.6 mm thick, 95% porosity, 20 pores per cm) and on PTFE (polytetrafluoroethylene) membranes (Pall Corporation PTF002LH0A-SAMP, 0.02 µm pore size, polypropylene backed). For characterization of coatings Si (100) pieces were also employed as substrates in addition to Ni foam and PTFE membranes, all coated simultaneously. In a previous work to study the use of MS to fabricate catalytic Co films^21^ we validated the use of different supports for characterization techniques and catalytic studies. The methodology allows the deposition of conformal and complete coatings with their microstructure and composition independent on the support^21^. Anyway after each deposition, samples grown in this work over different supports were characterized by scanning electron microscopy and energy dispersive X-ray spectroscopy to confirm microstructure and composition reproducibility. Before deposition the Ni foam was cut into ca. 0.5 × 0.5 cm^2^ pieces and grouped to be used in a small reactor. Each group was weighted before and after deposition to obtain the total mass of catalyst deposited. Before each synthesis the Ni foam pieces were washed in an ultrasonic bath successively with distilled water, ethanol/acetone (1:1), HCl 0.1 M and again distilled water, ethanol and acetone to finally be dried in air for 24 hours. No previous treatment was done on PTFE membranes. The Si substrates were cleaned with acetone and dried in a nitrogen flow.

### Catalyst characterization

X-ray diffraction measurements were performed using Cu Kα radiation in a Siemens D5000 diffractometer in a Bragg-Brentano configuration. Coatings grown on PTFE membranes were used for these measurements.

Scanning electron microscopy (SEM) was performed to study the morphology and microstructure of samples in a high resolution FEG microscope HITACHI S4800. Coatings were analysed directly on the substrates for top view observations. For cross section views, cleaved samples from coatings grown on Si were used. The thickness of the coatings was evaluated from the SEM cross-section measurements.

TEM (Transmission Electron Microscopy) and SAED (selected area electron diffraction) studies were performed on a FEI Tecnai G2 F30 FEG (field emission gun) microscope, equipped with a HAADF (High Angle Annular Dark Field) detector from Fischione Instruments. Images were obtained in TEM and STEM/HAADF (scanning TEM) mode at 300 kV. Once the thin films were grown on PTFE membranes these were first removed from the polypropylene back-support, fixed in a copper frame grid, and submitted to ion thinning from the back-side before analysis.

Proton-elastic back-scattering spectrometry (p-EBS) was used to determine the composition of the catalytic coatings as grown on Si substrates. This technique is well suited for characterization of coatings with simultaneous determination of Ru, Co and the light elements C, O and B^34^. Measurements were carried out at the National Center for Accelerators (CNA, Sevilla, Spain) using a 3MV tandem accelerator. The spectra were obtained with a 2 MeV H^+^ beam and passivated implanted planar silicon (PIPS) detector set at 165°. To obtain the thickness and composition of the films, spectra were simulated using the SIMNRA code^35^.

XPS spectra were recorded with a SPECS electron spectrometer equipped with a PHOIBOS 150 9MCD analyzer using Al Kα radiation with 35 eV pass energy at normal emission take off angle. The spectra were calibrated with the position of surface oxidized Co (2p_3/2_) at 780.9 eV, because of the superimposition of the C (1 s) level of adventitious carbon with the Ru 3d_3/2_ level of Ru.

### Catalytic tests

The Ni foam pieces were grouped (10 to 15 pieces), cleaned and weighted before and after deposition. The set of pieces, with the supported catalyst (1–6 mg), was placed at the bottom of a three necked heart-shaped flask. The flask was immersed in a water bath maintained at (25.0 ± 0.5) °C and connected to a gas burette^36^. The SB hydrolysis reaction started by injecting 38 mg of SB (Sigma Adrich 99%) dissolved in 1 ml of 4.5 wt% NaOH solution. The amount of generated hydrogen was determined by measuring the mass of water displaced in the gas burette as a function of time^36^. No additional stirring was used for the experiments, except for the stirring effect of the evolved hydrogen. The HGR (Hydrogen Generation Rate, (ml.min^−1^) was obtained from the slope of the plot of the volume of hydrogen evolved *vs* time in linear regime. Catalytic activity (expressed in ml.min^−1^.g_cat_^−1^) was obtained as the slope from the plot of HGR (ml.min^−1^) as a function of the mass of supported catalyst. Activity was also expressed per gram of total metal considering the composition of catalysts as determined by p-EBS spectrometry. Finally, to get the apparent activation energy, catalytic activities were measured for selected samples at different temperatures and the Arrhenius plot was analysed. Cycling experiments were also carried out. After each test, the supported catalyst was extracted from the reaction medium and thoroughly washed with distilled water and ethanol then it was dried during one day in atmospheric conditions before repeating the activity test. Additional cycling experiments were also carried out avoiding the extraction of the catalysts from the reactor by following a quick washing procedure as described when required.

## Electronic supplementary material

Supplementary information
