# Author Correction: Strong activation effect on a Ru-Co-C thin film catalyst for the hydrolysis of sodium borohydride

**DOI:** 10.1038/s41598-018-31701-1

**Published:** 2018-09-04

**Authors:** G. M. Arzac, M. Paladini, V. Godinho, A. M. Beltrán, M. C. Jiménez de Haro, A. Fernández

**Affiliations:** 1 Instituto de Ciencia de Materiales de Sevilla (CSIC-Univ. Sevilla), Avda. Américo Vespucio 49, 41092 Sevilla, Spain; 2 Departamento de Ingeniería y Ciencia de los Materiales y del Transporte, Universidad de Sevilla, Escuela Politécnica Superior, Virgen de África 7, 41011 Sevilla, Spain

Correction to: *Scientific Reports* 10.1038/s41598-018-28032-6, published online 27 June 2018

The original version of this Article contained an error in the title of the paper, where “Ru-Co-C thin film catalyst” was incorrectly given as “ru-co-c thin film catalyst”. This has now been corrected in the PDF and HTML versions of the Article.

