# Supplementary material for: Strong activation effect on a Ru-Co-C thin film catalyst for the hydrolysis of sodium borohydride
Source: Sci Rep. 2018 Jun 27;8:9755. doi: 10.1038/s41598-018-28032-6 (PMC6021453; doi:10.1038/s41598-018-28032-6)
Supplement: Supplementary file 1 — Supplementary information [file 41598_2018_28032_MOESM1_ESM.pdf]

STRONG ACTIVATION EFFECT ON A Ru-Co-C THIN FILM CATALYST FOR THE HYDROLYSIS OF SODIUM BOROHYDRIDE

G.M. Arzac*, M. Paladini, V. Godinho, A.M. Beltrán[1], M.C. Jiménez de Haro, A. Fernández*

Instituto de Ciencia de Materiales de Sevilla (CSIC-Univ. Sevilla), Avda. Américo Vespucio 49, 41092-Sevilla, Spain.

[1] Departamento de Ingeniería y Ciencia de los Materiales y del Transporte, Universidad de Sevilla, Escuela Politécnica Superior, Virgen de África 7, 41011 Sevilla, Spain

* Corresponding authors: gisela@icmse.csic.es (G.M. Arzac), asuncion@icmse.csic.es (A. Fernández)

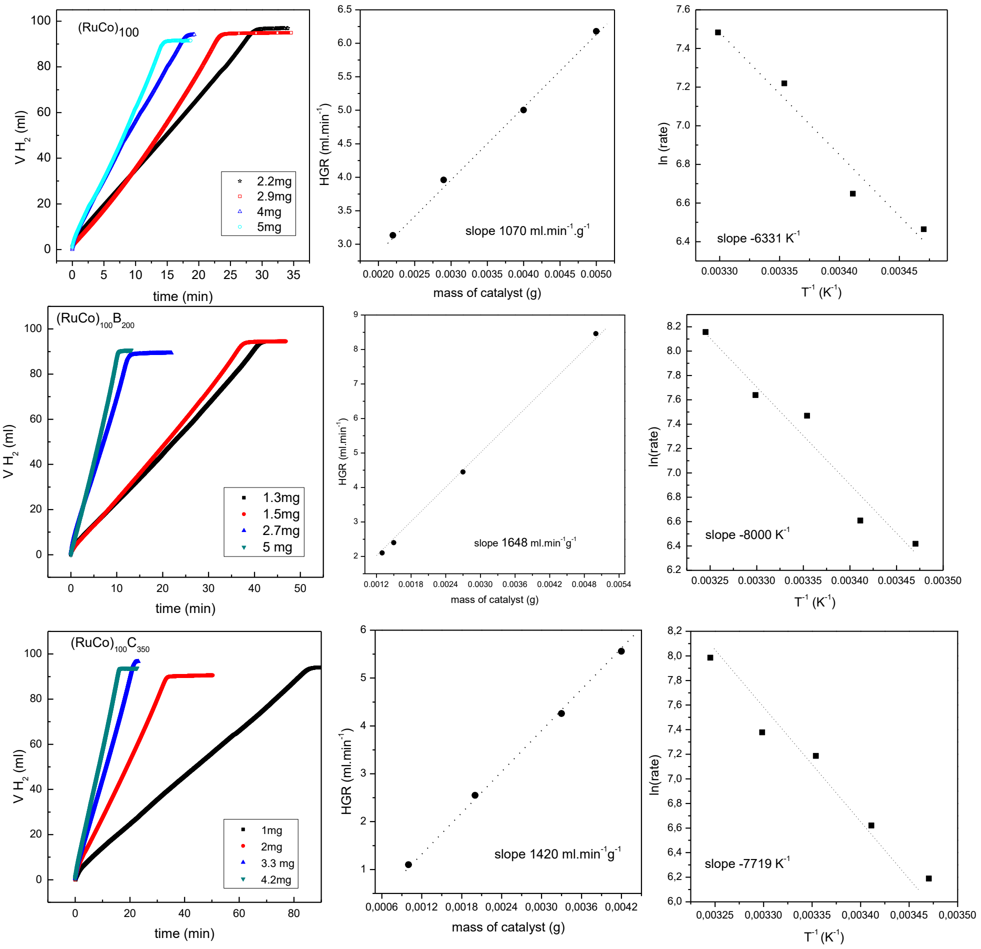

Figure S.1.- Hydrogen evolution curves, hydrogen generation rates as a function of the mass of catalyst and Arrhenius plots for the prepared catalysts

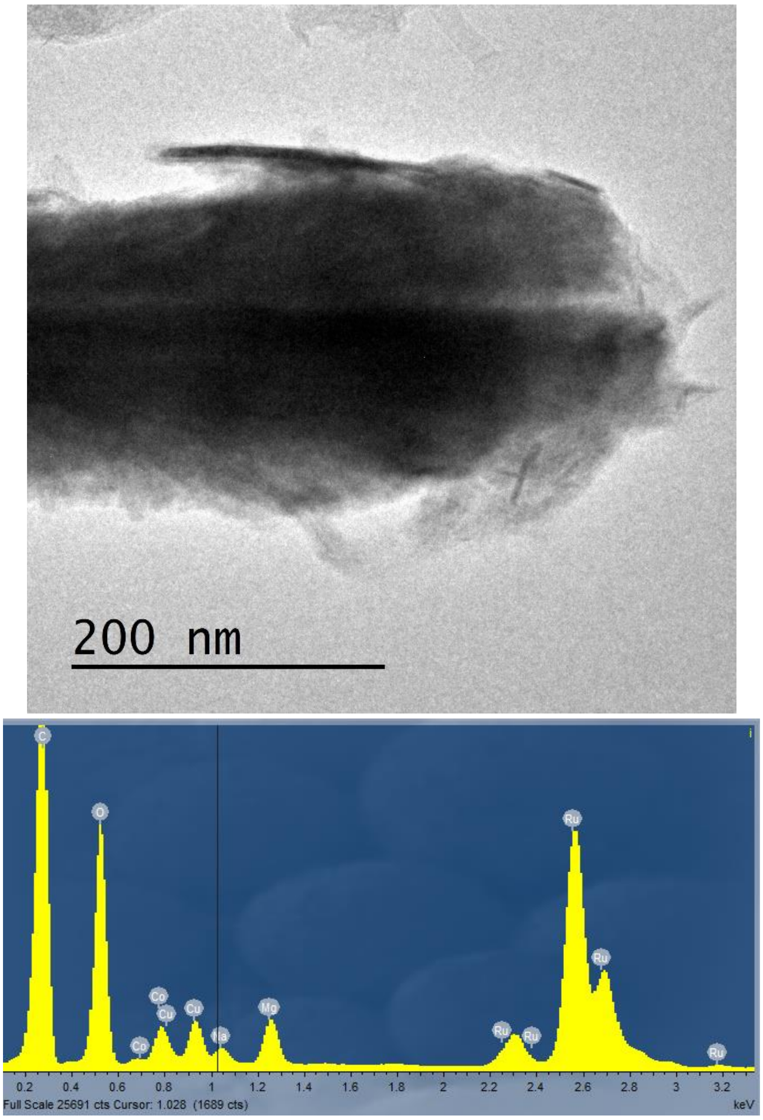

Figure S.2. Study of the brown reaction precipitates obtained from the supernatant washing solutions after reaction. Representative TEM image and EDX spectrum of a portion of thin film mechanically detached from the support during the reaction.
